# RBX1^+^ CAFs Drives Pancreatic Ductal Adenocarcinoma Progression Through Tenascin C Overexpression

**DOI:** 10.3390/cancers18061024

**Published:** 2026-03-22

**Authors:** Qinwen Zuo, Ziheng Wang, Chengxiao Yang, Binghang Yan, Jiaming Li, Mingkai Cui, Meng Cai, Hongze Chen, Xuewei Bai

**Affiliations:** 1 The Pancreatic Disease Diagnosis and Treatment Center, The First Affiliated Hospital of Harbin Medical University, Harbin 150001, China; 2 Key Laboratory of Hepatosplenic Surgery, Ministry of Education, The First Affiliated Hospital of Harbin Medical University, Harbin 150001, China

**Keywords:** pancreatic ductal adenocarcinoma, RBX1, cancer-associated fibroblasts, Tenascin C, tumor microenvironment

## Abstract

**Background:** Pancreatic ductal adenocarcinoma (PDAC) is characterized by a dense desmoplastic stroma that actively drives malignant progression. However, the specific contributions of E3 ubiquitin ligases within the cancer-associated fibroblast (CAFs) compartment to the PDAC landscape remain largely elusive. **Methods:** Pancreatic tissue samples were collected from the First Affiliated Hospital of Harbin Medical University. Gene expression was analyzed by RT-PCR, and single-cell RNA sequencing (scRNA-seq) data were integrated for cell subtype identification. Kaplan-Meier survival analysis assessed gene expression and survival. Pseudotime analysis and CellChat evaluated fibroblast transitions and intercellular communication. Cell lines were transfected with RBX1 siRNAs, and protein levels were measured by Western blotting. Proliferation was assessed using colony formation and EdU staining. Statistical analyses were performed using R (v4.4) and GraphPad Prism 8.0. **Results:** Thirteen E3 ubiquitin ligases were significantly upregulated in PDAC and correlated with unfavorable clinical outcomes. Among these, RBX1 was identified as a candidate preferentially expressed in CAF populations and strongly associated with poor prognosis. Single-cell transcriptomic profiling and pseudotime analysis further revealed that RBX1-positive CAFs were predominantly involved in extracellular matrix remodeling and pro-tumorigenic pathways. Functional assays demonstrated that silencing RBX1 markedly inhibited PAAD cell proliferation and tumor growth both in vitro and in xenograft models. Mechanistically, RBX1 was found to upregulate Tenascin C (TNC) expression, while ectopic overexpression of TNC partially rescued the growth suppression induced by RBX1 knockdown. **Conclusions:** Our findings suggest that RBX1 facilitates PDAC progression through a CAF-related mechanism and TNC regulation, positioning RBX1 as a potential therapeutic target for PDAC intervention.

## Simple Summary

Cancer-associated fibroblasts (CAFs) are important components of the tumor microenvironment and contribute to tumor progression, immune regulation, and resistance to therapy. However, the molecular mechanisms controlling CAF activity remain unclear. In this study, we examined the role of RBX1 in CAFs and its potential contribution to tumor development. By integrating single-cell RNA sequencing data with experimental validation, we found that RBX1 is highly expressed in CAF populations and is associated with pathways related to tumor promotion. Functional analyses suggest that RBX1 may influence CAF activity and support tumor progression. These findings provide new insight into CAF regulation and indicate that RBX1-related pathways may represent potential targets for future cancer therapy.

## 1. Introduction

Pancreatic ductal adenocarcinoma (PDAC), a major subtype of pancreatic adenocarcinoma (PAAD), continues to be one of the most aggressive and lethal cancers, with five-year survival rates still remaining very low despite recent advances in treatment [1,2]. The poor prognosis is largely due to late diagnosis, resistance to treatments, and the inherent complexity of the disease [3,4]. A key feature of PDAC is its large stromal compartment, which often exceeds the volume of tumor cells. Cancer-associated fibroblasts (CAFs) are a major component of this stroma [5], and it plays a crucial role in tumor development by affecting processes such as matrix remodeling, immune response modulation, angiogenesis, and therapy resistance [6,7,8,9].

Emerging single-cell analyses have revealed that CAFs are not a homogeneous group but consist of distinct subpopulations, each with specific transcriptional and functional characteristics [10,11,12]. However, the underlying factors that drive CAF diversity and their influence on PDAC progression are still not well understood. One important regulatory mechanism in this context is protein ubiquitination, which governs protein degradation and cellular signaling [13,14,15]. E3 ubiquitin ligases, responsible for substrate specificity in the ubiquitination process, are known to regulate various cellular processes. Altered expression of these ligases has been associated with cancer progression, metastasis, and immune escape [16,17]. While the role of E3 ligases in PDAC tumor cells has been extensively studied, their involvement in stromal fibroblasts remains less explored.

In this study, we sought to identify E3 ligases with potential prognostic value in PDAC. By analyzing bulk transcriptomic data alongside survival outcomes and integrating single-cell RNA sequencing, we identified a significant E3 ligase enriched in CAFs that is linked to poor clinical prognosis. Our experimental data show that this ligase promotes PDAC cell growth both in vitro and in vivo. Additionally, Tenascin C (TNC) was identified as a key downstream target, indicating a critical RBX1-TNC signaling axis that facilitates PDAC progression. These findings suggest that targeting stromal components, such as RBX1, could offer new therapeutic opportunities in PDAC treatment.

## 2. Materials and Methods

### 2.1. Sample Collection and Patient Information

PDAC tissues and matched adjacent normal pancreatic tissues were obtained from the Pancreatic Disease Diagnosis and Treatment Center of the First Affiliated Hospital of Harbin Medical University. Adjacent normal tissues were collected approximately 1 cm away from the tumor margin, and their lack of tumor infiltration was confirmed by histopathological examination. The study protocol was reviewed and approved by the Institutional Ethics Committee of the First Affiliated Hospital of Harbin Medical University, and written informed consent was obtained from all patients prior to sample collection. A total of 30 tissue specimens, comprising 15 tumor samples and 15 paired adjacent tissues, were collected for subsequent experimental analyses.

### 2.2. RT-PCR

Total RNA was extracted from tissues and cells using the FastPure RNA Kit (Axygen, Hangzhou, China) according to the manufacturer’s protocols. Subsequently, cDNA was synthesized using the HiScript^®^ III RT SuperMix for qPCR (TOYOBO, Shanghai, China). Quantitative real-time PCR (qPCR) was then performed using ChamQ SYBR qPCR Master Mix (Sangon Biotech, Shanghai, China) to quantify target gene expression, with β-Actin serving as the internal control for normalization.

### 2.3. Single-Cell RNA Sequencing (scRNA-Seq)

Public scRNA-seq datasets utilized in this study were retrieved from multiple authoritative repositories. Specifically, datasets GSE197177 (Normal = 1, Tumor = 6), GSE212966 (Normal = 6, Tumor = 6), and GSE217845 (Tumor = 9) were sourced from the Gene Expression Omnibus (GEO) database. For the analysis of normalized gene expression profiles and microarray cohorts of PDAC, publicly available data were integrated from The Cancer Genome Atlas (TCGA) data portal and the GEPIA database.

For quality control, cells with 200 < nFeature_RNA < 2500 and percent.mt < 25% were retained for downstream analysis. To mitigate batch effects and prevent individual heterogeneity from biasing the downstream analysis, the Harmony algorithm was employed for cross-sample integration. For dimensionality reduction and cell subtype identification, the top 3000 highly variable genes (HVGs) were identified using the FindVariableFeatures function, followed by data scaling. Principal component analysis (PCA) was subsequently performed based on these HVGs. A nearest-neighbor graph was constructed using the first 50 principal components via the FindNeighbors function, and cell clustering was executed using the FindCluster algorithm. The resulting clusters were visualized using Uniform Manifold Approximation and Projection (UMAP). Furthermore, gene signature scoring was performed to characterize the identified cell lineages. Differentially expressed genes (DEGs) among clusters were identified using the FindAllMarkers function with the following criteria: min.pct = 0.15, logfc.threshold = 0.15, and only.pos = TRUE. *p*-values and adjusted *p*-values were determined using the Wilcoxon rank-sum test with Benjamini-Hochberg (BH) correction.

### 2.4. Survival Analysis

Kaplan-Meier survival analysis was performed to evaluate the association between gene expression levels and overall survival of patients. For each key gene, patients were stratified into high- and low-expression groups based on the median expression value. Differences in survival curves between groups were compared using the log-rank test.

### 2.5. Pseudotime Analysis

To elucidate the dynamic evolutionary transition from quiescent fibroblasts to CAFs in PDAC, pseudotime trajectory analysis was performed using the Monocle3 R package (v1.3.1). During the data preprocessing stage, PCA was executed using the preprocess_cds function with the dimensionality set to 50. Subsequently, non-linear dimensionality reduction was performed via the UMAP algorithm, and the underlying cellular differentiation structure was inferred using the learn_graph function. To simulate the biological progression from a physiological state to a pro-tumorigenic phenotype, fibroblasts derived from normal pancreatic tissues were designated as the “root” of the trajectory using the order_cells function. Finally, the dynamic expression patterns of key CAF markers, including ACTA2, COL1A1, and POSTN, were analyzed along the pseudotime axis using the plot_genes_in_pseudotime function, with gene expression trends visualized through Loess-smoothed curves.

### 2.6. CellChat Analysis

To systematically characterize the signaling crosstalk between RBX1^+^ CAFs and other constituent cells within the PDAC microenvironment, intercellular communication analysis was performed using the CellChat R package (v1.6.1). Initially, a CellChat object was constructed based on the log-normalized expression matrix comprising 11 distinct cell populations. The “Secreted Signaling” category from the CellChatDB.human database was selected as the reference ligand-receptor interaction library. Communication probabilities among cell groups were inferred using the computeCommunProb function, which is based on the law of mass action. To ensure the robustness of the inferred networks, signaling pathways supported by fewer than 10 cells were excluded from the analysis via the filterCommunication function. The aggregate number and functional strength of interactions were calculated using the aggregateNet function and visualized through circle plots and heatmaps. To identify key signaling hubs, network centrality scores, including outdegree and indegree, were computed using the netAnalysis_computeCentrality function.

### 2.7. Cell Culture

Human pancreatic cancer (PC) cell lines, including MIA PaCa-2 and PANC-1, were purchased from the American Type Culture Collection (ATCC). These cell lines were maintained in Dulbecco’s Modified Eagle Medium (DMEM) (gibco, Carlsbad, CA, USA) supplemented with 10% fetal bovine serum (FBS) (Sangon Biotech, Shanghai, China) and 100 units/mL penicillin/streptomycin. Normal fibroblasts (NFs) and CAFs were isolated from paired adjacent normal tissues and tumor tissues obtained from surgical specimens using a tissue explant adhesion method. All tissue samples were collected immediately after surgical resection and transferred into sterile phosphate-buffered saline (PBS) supplemented with 1% penicillin-streptomycin. The tissues were washed three times with PBS to remove blood and debris. Necrotic tissues, visible vessels, and adipose tissues were carefully removed under sterile conditions. The remaining tissues were minced into small fragments of approximately 1 mm^3^ using sterile scissors. The tissue fragments were evenly placed onto the bottom of culture dishes and allowed to adhere for 2–5 min under sterile conditions. Subsequently, DMEM/F-12 medium (gibco, California, USA) supplemented with 20% FBS, and 1% penicillin-streptomycin was gently added to the dishes to avoid floating of the tissue fragments. The culture medium was replaced every 2–3 days to remove unattached cells and tissue debris. Fibroblast-like cells were observed to migrate from the tissue explants within 3–5 days. When the cells reached approximately 80–90% confluence, they were digested with 0.25% trypsin-EDTA and passaged at a ratio of 1:3. The cells were then routinely cultured in DMEM supplemented with 10% FBS. All cells were incubated at 37 °C in a humidified atmosphere containing 5% CO_2._

### 2.8. Transfection

Cells were transfected with small interfering RNAs (siRNAs) targeting RBX1 using Lipofectamine RNAiMAX (Invitrogen, Shanghai, China) according to the manufacturer’s instructions. Forty-eight hours after transfection, cells were harvested for subsequent experiments. Three siRNAs targeting RBX1 were designed, and the most efficient sequence was used for subsequent stable knockdown construction.

For rescue experiments, the TNC overexpression plasmid was purchased from Sangon Biotech (Shanghai, China). CAFs were seeded into six-well plates and transfected with the TNC overexpression plasmid using Lipofectamine 3000 (Sangon Biotech, Shanghai, China) according to the manufacturer’s protocol. Briefly, plasmid DNA and Lipofectamine 3000 reagent were diluted in Opti-MEM medium (gibco, California, USA), mixed gently, and incubated for 15 min at room temperature to form DNA-lipid complexes. The complexes were then added to the cells and incubated for 6 h, after which the medium was replaced with fresh complete culture medium. Cells were collected 48 h after transfection for subsequent analyses.

### 2.9. Generation of Stable RBX1 Knockdown CAFs

To establish CAFs with stable RBX1 knockdown, three small interfering RNAs targeting RBX1 (si-RBX1#1, si-RBX1#2, and si-RBX1#3) were initially designed and evaluated. The sequences were as follows: si-RBX1#1: 5′-GCAUAGAAUGUCAAGCUAA-3′; si-RBX1#2: 5′-GCUACUUCAGAAGAGUGUA-3′; and si-RBX1#3: 5′-AGGUGUGUCCAUUGGACAA-3′. Based on the knockdown efficiency determined by qRT-PCR and Western blot analysis, si-RBX1#3 exhibited the most effective silencing and was therefore selected for the construction of a stable knockdown system.

The sequence corresponding to si-RBX1#3 was cloned into a lentiviral vector containing a puromycin resistance cassette to generate shRBX1 lentiviral plasmids. Lentiviral particles were produced by co-transfecting HEK293T cells with the shRBX1 plasmid together with the packaging plasmids psPAX2 and pMD2.G using Lipofectamine 3000 according to the manufacturer’s instructions.

Forty-eight hours after transfection, viral supernatants were collected, filtered through a 0.45 μm membrane, and used to infect CAFs in the presence of 8 μg/mL Polybrene. After 48 h of infection, cells were subjected to puromycin selection (2 μg/mL) for 6 days to generate stable RBX1-knockdown CAFs (sh-RBX1). CAFs transduced with a non-targeting control vector were used as negative controls (sh-NC).

The knockdown efficiency of RBX1 in the stable cell lines was verified by qRT-PCR and Western blot analysis prior to subsequent experiments.

### 2.10. Western Blotting

Total protein was extracted from cell lysates using RIPA buffer (Solarbio, Beijing, China). Protein samples were denatured by boiling in SDS loading buffer for 5–10 min at 95 °C prior to separation by 10% SDS-PAGE (Epizyme, Shanghai, China), with 30 μg of total protein loaded per lane. Separated proteins were transferred to PVDF membranes (Sigma-Aldrich, St. Louis, MO, USA). Membranes were blocked with 3% bovine serum albumin (BSA) for 30min. Subsequently, membranes were incubated with primary antibodies for 12–18 h at 4 °C, followed by incubation with appropriate secondary antibodies for 1–4 h. Protein bands were visualized using chemiluminescence detection.

### 2.11. Colony Formation Assay

Five hundred PC cells were plated on six-well plates. CAFs were plated in Transwell inserts and co-cultured with PC cells to allow paracrine signaling without direct cell contact. The co-culture system was maintained in complete DMEM containing 10% FBS at 37 °C in a humidified incubator (Thermo Fisher Scientific, Waltham, MA, USA) with 5% CO_2_. After 14 days of culture, the medium was removed and the cells were fixed with 4% paraformaldehyde for 15 min and stained with 2% crystal violet for 30 min. The wells were then cleaned, allowed to dry, and photographed. ImageJ 4.1 software was used to quantify the number of clones.

### 2.12. 5-Ethynyl-2-deoxyuridine (EdU) Staining

EdU incorporation assays were conducted using the EdU Cell Proliferation Kit 555 (Beyotime, Shanghai, China) according to the manufacturer’s instructions. Cells were incubated with EdU (10 μM) for two hours at 37 °C. Subsequently, the plates were rinsed with PBS, fixed with 4% paraformaldehyde for 10 min, and stained with DAPI for 15 min. EdU-positive cells were visualized using fluorescence microscopy (Olympus, Tokyo, Japan), and the percentage of EdU-positive cells was quantified with ImageJ 4.1.

### 2.13. Histological and Immunohistochemical Analysis

Pancreatic tissue specimens were fixed in 4% paraformaldehyde for 24 h, followed by dehydration, paraffin embedding, and serial sectioning into slices. Hematoxylin and Eosin (H&E) staining was performed to evaluate pathological morphology. For immunohistochemical (IHC) analysis, paraffin-embedded sections were deparaffinized, rehydrated, and subjected to heat-induced antigen retrieval in sodium citrate buffer (pH 6.0). Endogenous peroxidase activity was quenched with 3% H_2_O_2_, followed by blocking with 5% bovine serum albumin (BSA) for 30 min. Sections were incubated overnight at 4 °C with primary antibodies. On the following day, slides were incubated with horseradish peroxidase (HRP)-conjugated secondary antibodies for 1 h at room temperature. Signals were visualized using a DAB substrate kit and counterstained with hematoxylin.

### 2.14. Animal Experiments

C57 mice (female, 6 weeks old) were purchased from Beijing Vital River Laboratory Animal Technology Co., Ltd. (Beijing, China). KPC mice (female, 6 weeks old) were purchased from Cyagen Biosciences Inc. (Suzhou, China) Age- and sex-matched wild-type C57 mice and KPC mice were used as controls. Before the injection procedure, the mice were anesthetized with 2.5% isoflurane, ensuring an adequate level of anesthesia as confirmed by the absence of reflexes and steady respiration. Tumor cells were either injected subcutaneously or at an orthotopic site, depending on the specific experimental setup. The injection site was thoroughly cleaned using 70% ethanol prior to the procedure. A sterile syringe containing 100 μL of the tumor cell suspension was used for the injection into the abdominal wall or other selected site. Following the injection, the area was disinfected again with alcohol, and the mice were returned to their cages for recovery and monitoring. Following the injection, tumor growth was monitored weekly. Tumor size was assessed by measuring the largest (D) and smallest (d) diameters with a caliper. Tumor volume (V) was estimated using the formula V = (π/6) × D × d^2^. Additionally, the mice were observed for changes in body weight, tumor growth progression, and overall health. All animals were housed in a specific pathogen-free environment with controlled conditions, including a constant temperature (22 ± 2 °C), 12-h light/dark cycle and 50 ± 5% humidity. The sterile drinking water was replaced twice a week. The above animals were commercially available and acclimated for at least one week before the experiment started. The laboratory management personnel allocated the mice and cage placements using a random grouping method, thereby ensuring single blinding for the experiment operators.

### 2.15. Statistical Analysis

Statistical analyses were performed as described in Seurat (v5.2.0), R (v4.4) and GraphPad Prism v.8.0 software. Comparisons between two groups were performed using unpaired *t*-tests, while multiple group comparisons were analyzed using one-way ANOVA followed by least significant difference (LSD) post hoc testing. Data are presented as the mean ± standard error of the mean (SEM). A *p*-value < 0.05 was considered statistically significant.

## 3. Results

### 3.1. Analysis of E3 Ligases and Their Prognostic Value in PAAD

**Figure 1:**
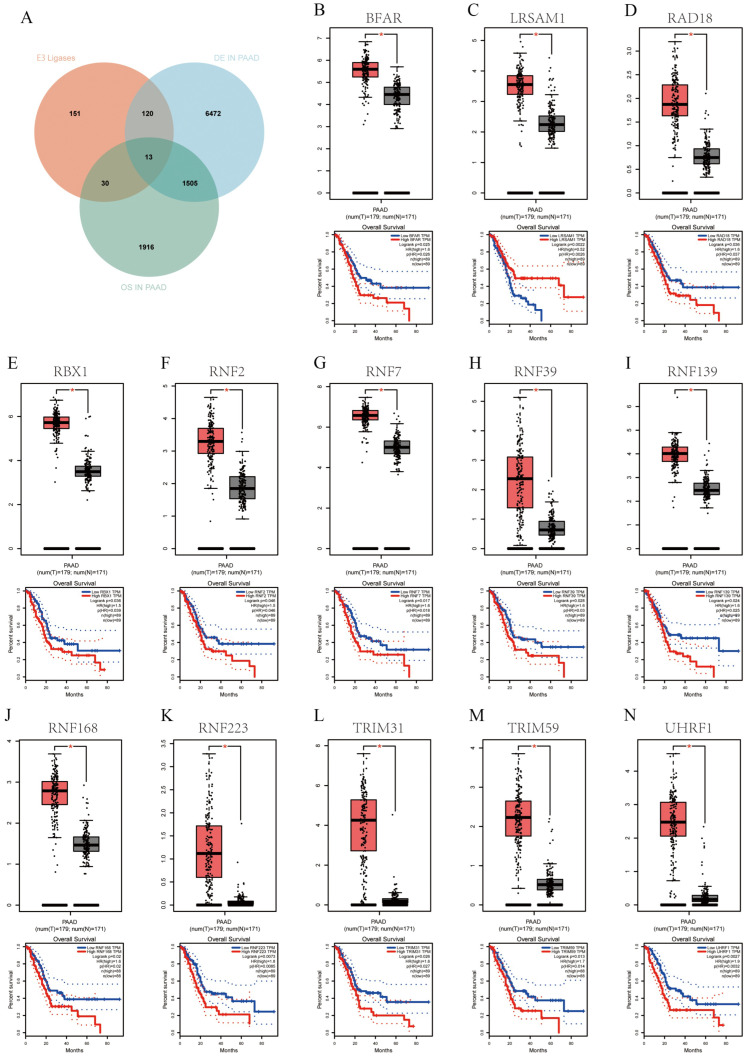
Gene expression and OS in PAAD. (**A**) Venn diagram of the overlap between three gene categories. (**B**–**N**) Box plots indicating significant differential expression of each gene in PAAD and patients and Kaplan-Meier Survival Curves with certain genes correlating with better or worse OS depending on their expression levels. *, *p* < 0.05.

To identify E3 ubiquitin ligases with potential prognostic significance in PAAD, we integrated differential expression profiling with overall survival (OS) analysis. By cross-referencing the E3 ligase gene library, PAAD-associated DEGs, and survival-correlated genes, we identified 13 overlapping candidates: BFAR, LRSAM1, RAD18, RBX1, RNF2, RNF7, RNF39, RNF139, RNF168, RNF223, TRIM31, TRIM59, and UHRF1 (Figure 1A). Expression profiling confirmed that all 13 genes were markedly elevated in tumor tissues compared with normal pancreatic samples (*p* < 0.05). Kaplan-Meier survival analysis further demonstrated that high expression of each candidate gene was significantly associated with shortened overall survival in PAAD patients. Functional enrichment analysis revealed that these candidates were predominantly enriched in molecular functions and biological processes such as metal ion binding, cation binding, protein ubiquitination, and protein modification by small protein conjugation (Figure S1A). Collectively, these findings indicate that multiple E3 ubiquitin ligases are aberrantly upregulated in PAAD and are closely linked to adverse clinical outcomes. This expression pattern underscores the potential role of dysregulated ubiquitination pathways in driving disease progression and provides a robust rationale for further functional investigation of these selected candidates (Figure 1B–N).

### 3.2. Single-Cell Transcriptomic Landscape of Pancreatic Tissues Across Histological Subtypes

**Figure 2:**
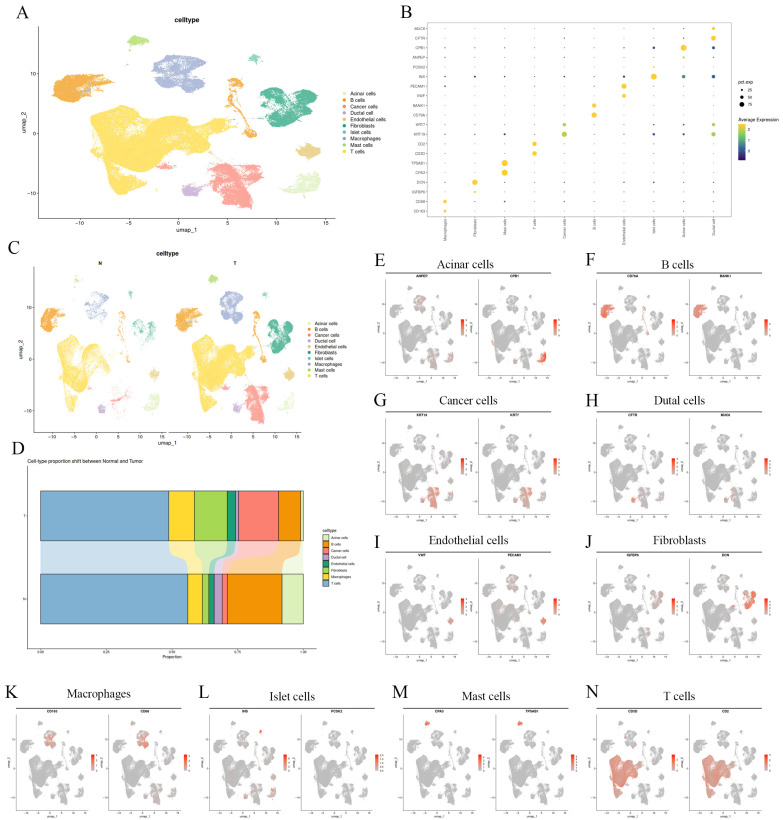
Single-cell transcriptomic landscape of pancreatic tissues across histological subtypes. (**A**) Uniform manifold approximation and projection (UMAP) plot showing the clustering of all cells into ten major cell types. (**B**) Dot plot illustrating canonical marker gene expression across identified cell types. (**C**) UMAP plots of Normal (N) and Tumor (T) samples colored by cell type, highlighting differences in cellular composition. (**D**) Histograms of the percentages of various types of cells in normal and pancreatic tumor tissues. (**E**–**N**) Feature plots showing the expression of representative marker genes projected onto the UMAP embedding, highlighting the spatial localization of distinct cell types.

To explore the cellular diversity within the pancreatic microenvironment, we analyzed scRNA-seq data derived from normal and tumor samples. After quality control and filtering, a total of 240,360 cells were retained for downstream analysis. Following rigorous quality control and unsupervised clustering analysis, ten principal cell populations were resolved. These included acinar cells, ductal cells, malignant epithelial cells, endothelial cells, fibroblasts, macrophages, T cells, B cells, mast cells, and islet cells (Figure 2A). These clusters were clearly separated in the UMAP space, indicating highly distinct transcriptional programs across different cell lineages. Cell identities were assigned based on the expression of established lineage-specific marker genes (Figure 2B). As anticipated, acinar cells were characterized by the expression of ANPEP and CPB1, whereas epithelial populations—comprising both ductal and malignant cells—exhibited high levels of KRT7 and KRT19. Endothelial cells showed enrichment for PECAM1 and VWF, fibroblasts expressed DCN, macrophages were marked by CD68 and CD163, and lymphocyte subsets were delineated by CD3D (T cells) and CD79A (B cells). A comparison between normal and tumor specimens revealed profound shifts in cellular composition (Figure 2C). Normal pancreatic tissue was predominantly composed of acinar and ductal cells. In contrast, tumor samples exhibited a prominent expansion of malignant epithelial cells, accompanied by a markedly increased representation of fibroblasts and immune cells. Notably, the fraction of acinar cells was precipitously reduced in tumor tissues, a finding consistent with the extensive tissue remodeling that occurs during malignant transformation (Figure 2D). Feature plot visualizations further corroborated the cluster annotations. Canonical markers such as PRSS1 and CPA1 (acinar), KRT7 and CFTR (ductal), and KRT8 and KRT18 (malignant epithelial cells) displayed spatially restricted expression patterns corresponding to specific clusters. Similarly, immune and stromal markers—including CD3D (T cells), CD79A (B cells), CD68 (macrophages), and DCN (fibroblasts)—were confined to discrete regions within the UMAP projection (Figure 2E–N). Collectively, these results demonstrate a robust cell-type annotation framework and underscore the extensive remodeling of the tumor microenvironment (TME) in PAAD.

### 3.3. Fibroblast-Specific Expression of E3 Ligases and Their Clinical Significance in PAAD

**Figure 3:**
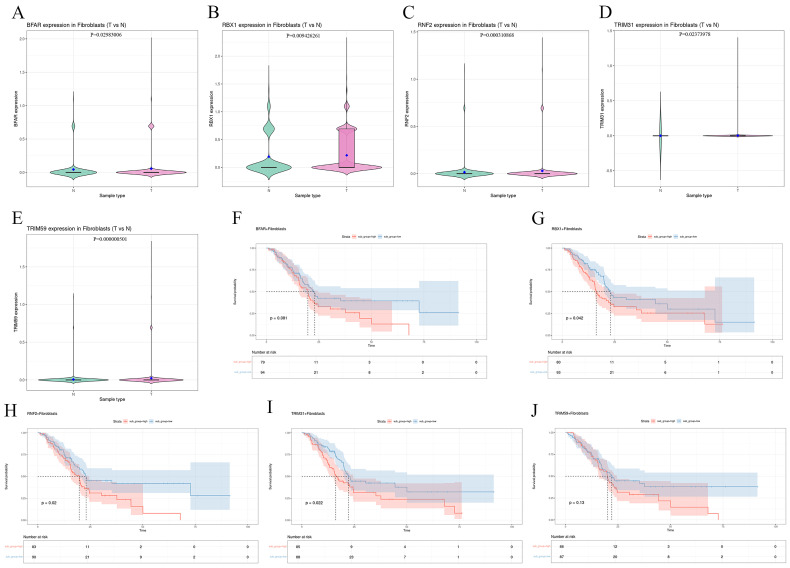
A comprehensive analysis of E3 ligase expression levels in fibroblasts from tumor and normal tissues. (**A**–**E**) Violin plots show the expression differences in E3 ligases with significant differential expression in PAAD tumor (T) and normal (N) fibroblasts. (**F**–**J**) Kaplan-Meier survival curves assessing the association between the expression of these genes in fibroblasts and patient survival.

To determine whether the candidate E3 ligases exhibit preferential dysregulation within the stromal compartment, we compared their expression profiles between tumor-associated and normal-derived fibroblast populations. Violin plot analysis demonstrated that a subset of candidates—specifically BFAR, RBX1, RNF2, TRIM31, and TRIM59—was significantly upregulated in CAFs, whereas the remaining genes showed no discernable differences (Figure 3A–E). The remaining genes have been moved to Figure S1B–I. We further investigated whether the fibroblast-enriched expression of these five genes possessed prognostic value. Kaplan-Meier survival analysis revealed that elevated fibroblast-associated levels of RBX1, RNF2, and TRIM31 were significantly correlated with truncated overall survival in PAAD patients. In contrast, no statistically significant associations were observed for BFAR or TRIM59. These findings indicate that while several E3 ubiquitin ligases are overexpressed in the stroma, only RBX1, RNF2, and TRIM31 are robustly linked to adverse clinical outcomes. This selective enrichment and its correlation with poor prognosis highlight their potential roles as key regulators of stromal-driven disease progression (Figure 3F–J). Among the three candidates associated with poor prognosis (RBX1, RNF2, and TRIM31), RBX1 exhibited the highest fold change in differential expression between CAFs and normal fibroblasts. Therefore, RBX1 was selected as the primary candidate for subsequent functional and mechanistic investigations.

### 3.4. Intercellular Interactions, Cell Type-Specific Signaling Pathways, and Immune Microenvironment Dynamics Associated with RBX1^–^CAFs

**Figure 4:**
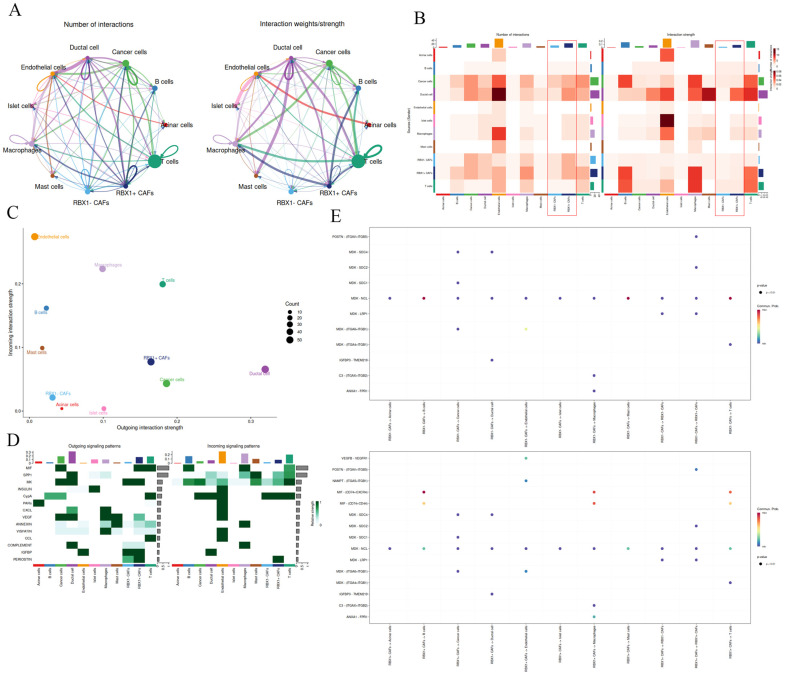
Analysis of the strength of interactions between cells and the signaling patterns of various cell types, including cancer cells and stromal cells. (**A**) Number of interactions and Interaction weights/strength among each cell type. (**B**) Number of interactions and interactions strength of all cell types. The red boxed regions highlight the interaction profiles associated with RBX1^+^ CAFs and RBX1^−^ CAFs. (**C**) Global analysis of cell-cell communication strength among distinct cell populations. (**D**) Outgoing signaling patterns and incoming signaling patterns of all cell types. (**E**) Dot plot showing clustering based on the interaction strength across different cell types.

To gain deeper insight into intercellular signaling within the pancreatic tumor TME, we analyzed cell–cell interactions, with a particular focus on the roles of CAFs and RBX1 in shaping tumor progression. Interaction network analysis showed that RBX1^+^ CAFs exhibited the broadest and strongest interactions with multiple cell types, including cancer cells, endothelial cells, and B cells (Figure 4A,B). The interaction matrix further supported this observation, indicating that RBX1^+^ CAFs—compared with RBX1^−^ CAFs—play a critical role in TME remodeling. Incoming and outgoing interaction strength analyses suggested that RBX1^+^ CAFs display high levels of both incoming and outgoing signaling (Figure 4C,D). In addition, robust ligand–receptor interactions were detected between MIF from RBX1^+^ CAFs and the CD74–CD44 complex on T cells, B cells, and macrophages (Figure 4E).

### 3.5. Pseudotime Mapping of RBX1^+^ CAF Activation in the Pancreatic TME

**Figure 5:**
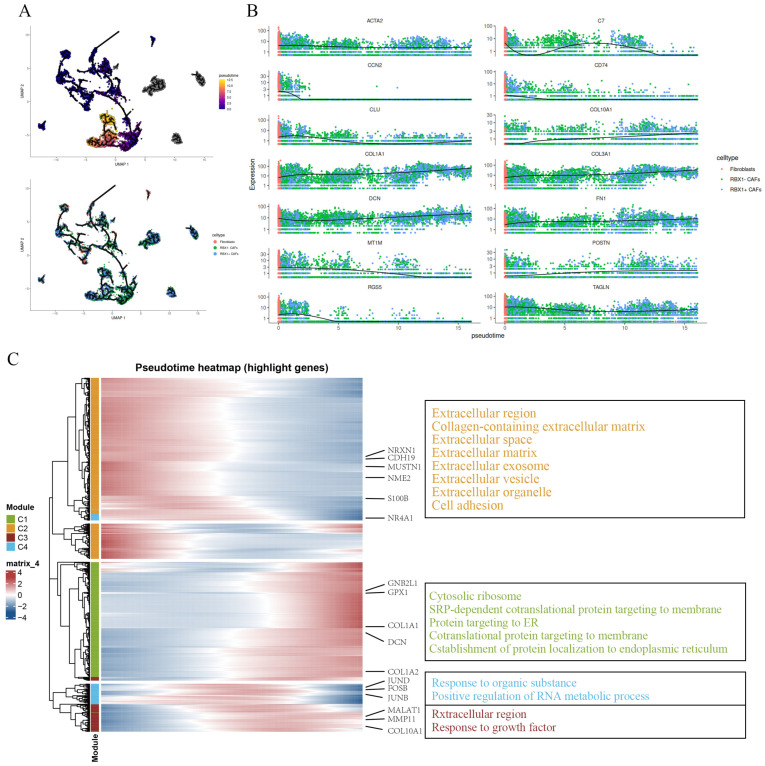
Pseudotime trajectories of gene expression in different cell states. (**A**) Pseudotime trajectory plot and gene expression trends. (**B**) Pseudotime projections showing the expression profiles of selected genes across the pseudotime trajectory. (**C**) Pseudotime heatmap showing the patterns of gene expression changes over pseudotime (**left**). The annotation box lists the functional categories of genes from different modules (**right**).

To explore how fibroblasts are activated over time in the pancreatic TME, we conducted pseudotime analysis using single-cell RNA sequencing data. This analysis revealed distinct cellular states along the pseudotime trajectory, with fibroblasts and RBX1 + CAFs showing differing expression patterns (Figure 5A). Genes related to fibrosis and extracellular matrix (ECM) remodeling, such as ACTA2, COL1A1, FN1, and POSTN, were notably upregulated along the pseudotime axis, particularly in RBX1 + CAFs, pointing to their key role in CAFs activation (Figure 5B). Further heatmap analysis of gene expression across pseudotime modules identified that fibroblast-related genes like COL1A1, DCN, COL1A2 were highly enriched in module C1, which is involved in ECM and collagen remodeling. Conversely, module C3, enriched with genes such as S100A8 and NRF2, was linked to immune responses and inflammation, indicating the active involvement of these pathways in the TME during fibroblast activation. Module C4, which includes genes like COL4A1, was associated with tumor growth factor signaling, emphasizing the role of RBX1 + CAFs in tumor-promoting signaling (Figure 5C). These observations highlight the critical role of RBX1 + CAFs in ECM remodeling and immune escape, with their activation potentially driving tumor progression and contributing to therapy resistance.

### 3.6. Progressive Upregulation of RBX1 in CAFs During PDAC Progression

**Figure 6:**
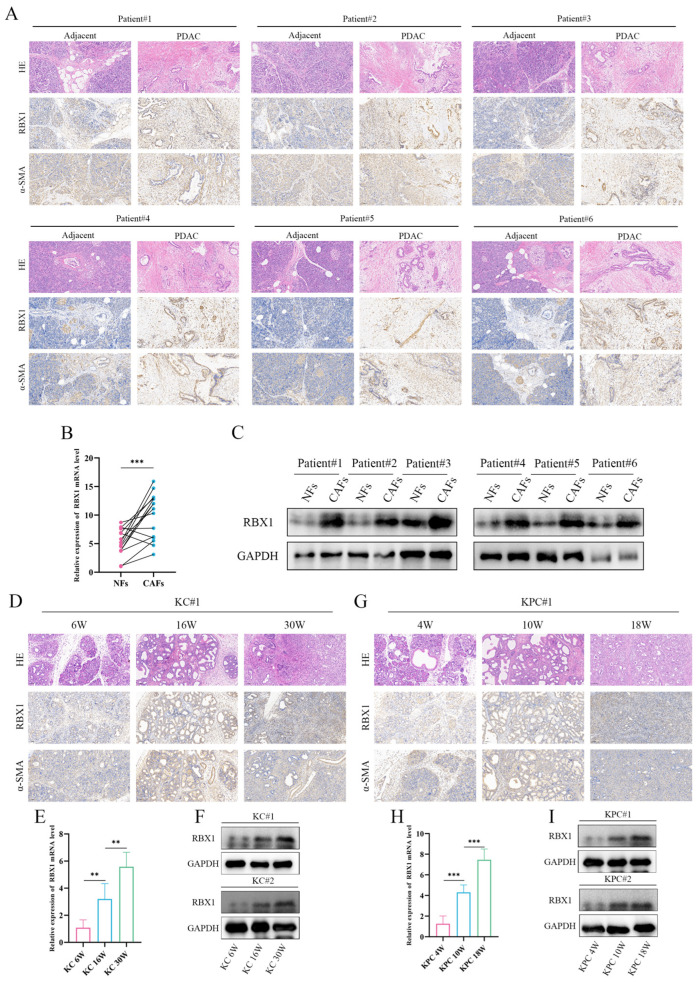
Differential expression of RBX1 in NFs and CAFs under different conditions. (**A**) Immunohistochemical staining of patient samples. (**B**) Quantitative analysis of RBX1 mRNA expression. (**C**) Western blot analysis of RBX1 expression. (**D**) Longitudinal study in KC mice. (**E**) Quantification of RBX1 mRNA expression in KC mice. (**F**) Western blot analysis of RBX1 in KC mice. (**G**) Longitudinal study in KPC mice. (**H**) Quantification of RBX1 mRNA expression in KPC mice. (**I**) Western blot analysis of RBX1 in KPC mice. ‘#’ indicates independent samples/replicates; **, *p* < 0.01; ***, *p* < 0.001.

Through histological examination and immunostaining, we observed a marked expansion of the stromal compartment in PDAC tissues. Immunohistochemical staining for RBX1 revealed that RBX1-positive cells were predominantly localized within the tumor stroma and were substantially increased in PDAC compared with adjacent tissues (Figure 6A). Consistently, α-SMA staining indicated a pronounced enrichment of CAFs in the PDAC stroma. Taken together, the spatial distribution of these signals suggests a significant accumulation of RBX1^+^ fibroblasts/CAFs within the PDAC TME (Figure 6A).

We next quantified RBX1 expression in NFs and CAFs. RT–qPCR analysis showed that RBX1 mRNA levels were significantly higher in CAFs than in NFs (*p* < 0.001), further supporting its potential role in the PDAC microenvironment (Figure 6B and Figure S1A,J). Western blot analysis of paired NFs and CAFs from six independent patients confirmed the upregulation of RBX1 at the protein level in CAFs, consistent with the mRNA results (Figure 6C).

In our study, each experimental group included 5 mice. The KC group was divided into three observation phases: early (6 weeks post-selection), middle (16 weeks post-selection), and late (30 weeks post-selection), during which time the progression of pancreatic intraepithelial neoplasia (PanIN) was closely monitored. For the KPC group, which carries a TP53 mutation with higher malignancy potential, the observation periods were set at 4, 10 and 18 weeks post-selection. In the KC mouse model, longitudinal profiling demonstrated a progressive increase in RBX1 expression over time. RBX1 was significantly upregulated at the late stage (30 weeks) compared with earlier time points (6 and 16 weeks), in parallel with tumor progression (Figure 6D and Figure S1K). Quantification of RBX1 mRNA revealed significant differences across time points (Figure 6E), and Western blotting further validated these temporal changes at the protein level (Figure 6F). A similar pattern was observed in the KPC mouse model, where RBX1 expression was significantly elevated at 18 weeks relative to earlier stages (4 and 10 weeks) (Figure 6G). mRNA quantification highlighted the dynamic changes in RBX1 during PDAC progression (Figure 6H), and Western blot analyses corroborated these findings at the protein level (Figure 6I). The remaining samples are shown in Figure S2.

Overall, these results indicate that RBX1 is progressively upregulated in PDAC tissues and fibroblasts, with particularly prominent elevation in CAFs, implicating RBX1^+^ fibroblasts/CAFs in PDAC progression. The consistent increase in RBX1 at both mRNA and protein levels across multiple patient-derived samples and animal models provides strong evidence for its involvement in the PDAC TME.

### 3.7. Investigating the Role of RBX1 in PAAD Proliferation and Tumorigenesis by Knocking Down the RBX1 Model

**Figure 7:**
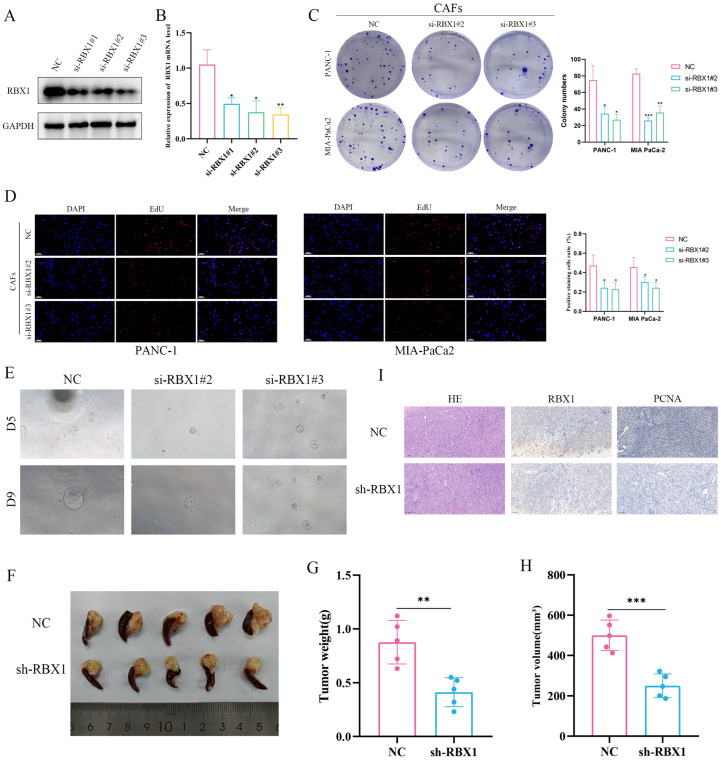
The role of RBX1 in PAAD cell proliferation and tumorigenesis. (**A**) Western blot analysis of RBX1 expression. (**B**) Quantification of RBX1 mRNA expression. (**C**) Colony formation assay. (**D**) EdU incorporation assay. (**E**) 3D organoid formation assay. (**F**) Tumor formation in nude mice. (**G**) Tumor weight formed after nude mice were injected with sh-RBX1 or NC cells. (**H**) Tumor volume formed after nude mice were injected with sh-RBX1 or NC cells. (**I**) Histological images of tumors in the NC group and sh-RBX1 group. ‘#’ indicates independent samples/replicates; *, *p* < 0.05; **, *p* < 0.01; ***, *p* < 0.001. The scale bar for EdU is 0.100 mm.

By establishing RBX1-knockdown CAFs, we systematically investigated the role of RBX1 in CAFs in regulating pancreatic adenocarcinoma (PAAD/PDAC) cell proliferation and tumorigenesis. Western blot and qRT–PCR analyses showed that RBX1 protein and mRNA levels were markedly reduced in CAFs transfected with si-RBX1 constructs (si-RBX1#1, si-RBX1#2, and si-RBX1#3) compared with the negative control (NC), confirming efficient knockdown (Figure 7A,B). In colony formation assays performed after co-culturing RBX1-depleted CAFs with PANC-1 and MIA PaCa-2 cells, both the number and size of colonies were significantly decreased, indicating impaired proliferative and clonogenic capacity of tumor cells (Figure 7C). Consistently, EdU incorporation assays revealed a significant reduction in the proportion of EdU-positive tumor cells in the RBX1-knockdown CAF group, further demonstrating that loss of RBX1 in CAFs suppresses tumor cell proliferation in both cell lines (Figure 7D). Moreover, in organoid culture experiments, organoids co-cultured with RBX1-knockdown CAFs exhibited a markedly reduced volume, suggesting that depletion of RBX1 in CAFs compromises the tumorigenic potential of PAAD/PDAC cells (Figure 7E). In vivo, stable RBX1 knockdown CAFs (sh-RBX1) were co-implanted with KPC cells into the pancreas of C57BL/6 mice using an orthotopic transplantation model. Tumors in the sh-RBX1 group were significantly smaller than those in the NC group (Figure 7F). Measurements of tumor weight and volume further confirmed that tumors from the sh-RBX1 group were lighter and displayed a significant reduction in volume (Figure 7G,H). Histological analyses showed decreased RBX1 expression and reduced staining of the proliferation marker PCNA in tumors from the sh-RBX1 group, supporting a diminished proliferative capacity upon RBX1 depletion (Figure 7I).

Collectively, these findings indicate that RBX1 in CAFs is critical for promoting PAAD/PDAC cell proliferation and in vivo tumorigenesis. Knockdown of RBX1 in CAFs significantly attenuated tumor-promoting phenotypes in both in vitro and in vivo models, suggesting that RBX1 may represent a potential therapeutic target for PDAC.

### 3.8. Expression Characteristics, Functional Enrichment Analysis, Correlation Analysis, and Prognostic Value of RBX1 and Its Related Genes in PAAD

**Figure 8:**
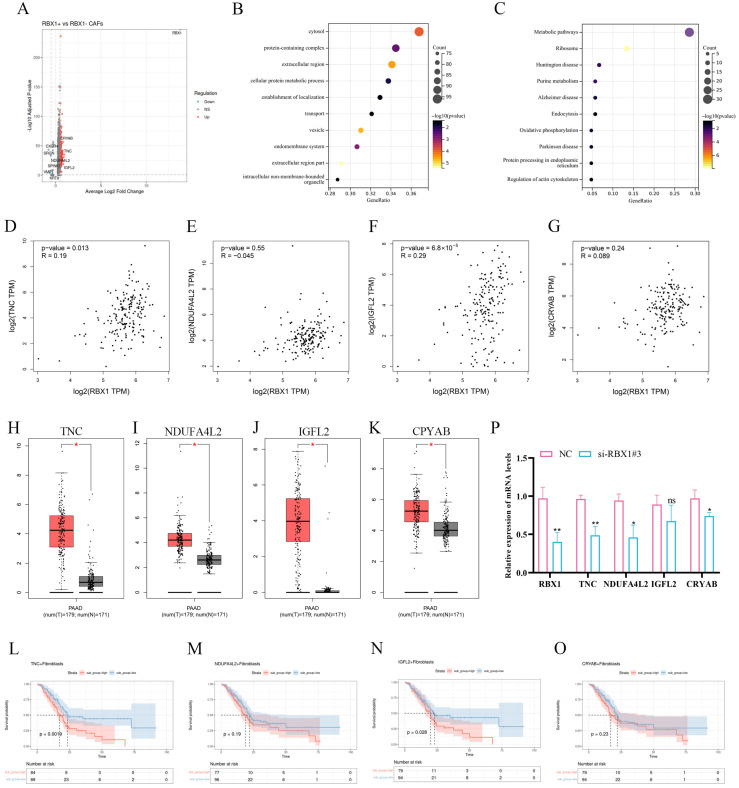
Integrated bioinformatics and experimental validation analyses reveal the oncogenic relevance of RBX1 and its associated genes in PAAD. (**A**) Volcano plot showing DEGs between RBX1-associated CAF-related groups. (**B**) GO enrichment analysis of DEGs. (**C**) KEGG pathway enrichment analysis of DEGs. (**D**–**G**) Pearson correlation analyses between RBX1 expression and candidate genes in PAAD samples. (**H**–**K**) Expression levels of TNC, NDUFA4L2, IGFL2, and CRYAB in PAAD tumor tissue and normal pancreatic tissues from TCGA dataset. (**L**–**O**) Kaplan-Meier survival analyses of OS in PAAD patients. (**P**) Relative mRNA expression levels of RBX1 and candidate genes following RBX1 knockdown compared with NC in vitro. ‘#’ indicates independent samples/replicates; *, *p* < 0.05; **, *p* < 0.01; ‘ns’ indicates ‘not significant’.

To elucidate the biological significance of RBX1^+^ CAFs in pancreatic adenocarcinoma (PAAD), we first performed differential expression analysis based on RBX1^+^ CAF-associated grouping. The volcano plot revealed a large number of significantly dysregulated genes, among which TNC, NDUFA4L2, IGFL2, and CRYAB were prominently upregulated (Figure 8A). These findings suggest that enrichment of RBX1^+^ CAFs is accompanied by extensive transcriptional remodeling within the TME.

Functional enrichment analysis further showed that RBX1^+^ CAF-associated DEGs were mainly enriched in cytosolic components, protein-containing complexes, extracellular regions, vesicular structures, and transport-related cellular architectures (Figure 8B). KEGG pathway analysis demonstrated significant enrichment in metabolic pathways, ribosome, oxidative phosphorylation, and protein processing in the endoplasmic reticulum (Figure 8C). Collectively, these data indicate that RBX1^+^ CAFs may participate in metabolic reprogramming and translational regulation, both of which are key features of tumor progression and stromal activation.

To explore potential downstream targets of RBX1, we conducted correlation analyses. RBX1 expression was significantly positively correlated with TNC, and showed an even stronger correlation with IGFL2 (Figure 8D,F). In contrast, no significant correlation was observed between RBX1 and NDUFA4L2 or CRYAB (Figure 8E,G). Analysis of the TCGA-PAAD dataset revealed that TNC, NDUFA4L2, IGFL2, and CRYAB were all significantly upregulated in tumor tissues compared with normal pancreatic tissues (Figure 8H–K). Kaplan–Meier survival analysis further showed that high TNC expression was significantly associated with poor overall survival (OS) (Figure 8L), and elevated IGFL2 expression also predicted an unfavorable prognosis (Figure 8N). By contrast, NDUFA4L2 and CRYAB did not reach statistical significance in survival analyses (Figure 8M,O).

To experimentally validate the regulatory role of RBX1, siRNA-mediated knockdown was performed in vitro. RBX1 silencing significantly reduced the mRNA levels of TNC, NDUFA4L2, and CRYAB, whereas IGFL2 expression was not significantly altered (Figure 8P).

### 3.9. CAFs-Derived RBX1 Promotes PAAD Progression via TNC

To test whether a downstream effector mediates the pro-tumorigenic function of RBX1 in CAFs, we conducted rescue experiments and chose TNC as a candidate based on differential expression, positive correlation with RBX1, and prognostic relevance.

**Figure 9:**
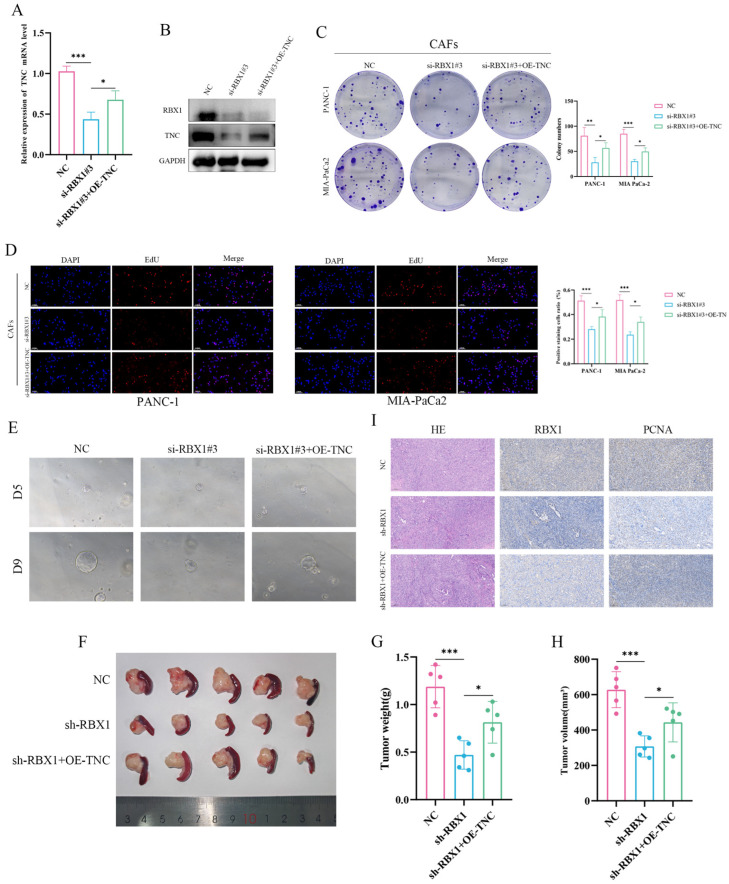
RBX1 promotes PAAD cell proliferation and tumor growth via TNC-dependent mechanisms in vitro and in vivo. (**A**) qRT-PCR analysis of RBX1 mRNA expression in PANC-1 and MIA PaCa-2 cells. (**B**) Western blot analysis of RBX1 and TNC protein levels. (**C**) Colony formation assays in PANC-1 and MIA PaCa-2 cells. (**D**) EdU incorporation assay to evaluate cell proliferation. (**E**) 3D organoid formation assay. (**F**) Representative images of xenograft tumors derived from nude mice injected with cells under the indicated treatments. (**G**) Tumor weight analysis of xenografts. (**H**) Tumor volume quantification of xenografts. (**I**) HE staining and IHC analysis of RBX1 and proliferation marker PCNA in xenograft tumor tissues. ‘#’ indicates independent samples/replicates; *, *p* < 0.05; **, *p* < 0.01; ***, *p* < 0.001. The scale bar for EdU is 0.100 mm.

qRT–PCR analysis showed that, compared with the negative control, si-RBX1#3 efficiently reduced RBX1 mRNA levels in CAFs (Figure 9A). Notably, co-transfection with a TNC overexpression plasmid did not restore RBX1 expression in CAFs, supporting the notion that TNC functions downstream of RBX1. Western blotting further confirmed that RBX1 knockdown in CAFs markedly decreased TNC protein levels, whereas TNC overexpression restored TNC expression without substantially affecting RBX1 levels (Figure 9B). These results suggest that RBX1 in CAFs positively regulates TNC expression, potentially at the transcriptional or post-transcriptional level.

Functionally, compared with the control group, RBX1-deficient CAFs significantly impaired colony formation of PANC-1 and MIA PaCa-2 cells (Figure 9C). Importantly, TNC overexpression partially reversed the reduction in clonogenicity caused by RBX1 knockdown, indicating that TNC mediates, at least in part, the pro-proliferative effect of RBX1. Consistently, EdU incorporation assays demonstrated a significant decrease in DNA synthesis upon RBX1 knockdown, while TNC overexpression partially restored the proportion of EdU-positive cells (Figure 9D). In addition, organoid culture assays showed that RBX1 depletion markedly suppressed organoid growth and size, and this inhibitory effect was partially alleviated by TNC overexpression (Figure 9E). Collectively, these in vitro findings support that RBX1 in CAFs promotes PAAD cell proliferation and three-dimensional growth, with TNC serving as an important downstream effector.

To further validate the pro-tumorigenic role of the RBX1–TNC axis in CAFs in vivo, we established an orthotopic pancreatic transplantation model in C57 mice. The NC group consisted of 10 mice, the sh-RBX1 group included 10 mice, and the sh-RBX1 + OE-TNC group contained 5 mice. Tumors formed in the sh-RBX1 group were significantly smaller than those in the control group (Figure 9F), and quantitative analyses confirmed markedly reduced tumor weight and volume (Figure 9G,H). Notably, TNC overexpression partially rescued the tumor growth suppression caused by RBX1 knockdown. HE staining revealed no obvious differences in necrosis, but sh-RBX1 tumors exhibited reduced cellular density. Immunohistochemistry confirmed efficient RBX1 silencing in vivo. Moreover, staining for the proliferation marker PCNA was significantly decreased in sh-RBX1 tumors and was partially restored in the sh-RBX1 + OE-TNC group (Figure 9I).

Taken together, the in vitro and in vivo rescue experiments consistently support RBX1 in CAFs as a promoter of PAAD tumor growth, and the tumor-promoting effects of RBX1 are partially reversible upon restoration of downstream TNC, highlighting the functional importance of the RBX1–TNC axis during PAAD progression.

## 4. Discussion

In this study, we aimed to uncover the molecular mechanisms by which RBX1, an E3 ubiquitin ligase, contributes to the progression of PAAD. By integrating multiple experimental and bioinformatics approaches, we provide compelling evidence that RBX1 plays a pivotal role in the TME, particularly in CAFs, and is a key regulator of tumor progression.

### 4.1. RBX1 as a Key Regulator in TME

Our analysis reveals that RBX1 is upregulated in tumor tissues compared to normal pancreatic tissues, and its expression is significantly correlated with poor OS in PAAD patients. This finding aligns with previous reports that have identified E3 ligases, including RBX1, as crucial regulators of cancer progression and metastasis [18,19,20]. This suggests that RBX1’s role extends beyond PAAD, supporting its broader significance in various cancers.

Moreover, single-cell RNA sequencing revealed a marked remodeling of the TME in PAAD, with a significant increase in the infiltration of fibroblasts and immune cells in tumor samples. The pronounced shift in cellular composition underscores the importance of stromal cells, especially CAFs, in modulating tumor progression. Our findings that RBX1 is particularly upregulated in CAFs and contributes to fibroblast-mediated tumor progression are consistent with recent literature, which highlights the central role of CAFs in shaping the cancer microenvironment [21,22].

### 4.2. E3 Ligases and Fibroblast-Specific Dysregulation

In our study, several E3 ligases, including RBX1, were found to be selectively activated in CAFs. Kaplan-Meier survival analyses further showed that elevated levels of RBX1, RNF2, and TRIM31 in fibroblasts were associated with poorer OS. These findings suggest that RBX1, alongside other E3 ligases, may serve as potential fibroblast-related prognostic biomarkers in PAAD. Similar conclusions were drawn in studies by Springer et al. [23], which proposed that E3 ligases can modulate stromal cell functions and contribute to tumor progression through ECM remodeling and immune modulation.

Additionally, pseudotime analysis revealed that RBX1 + CAFs exhibit distinct gene expression patterns related to ECM remodeling and immune evasion, including the upregulation of genes like ACTA2, COL1A1, and FN1. These findings suggest that RBX1 + CAFs not only contribute to fibrosis but also influence immune cell infiltration and tumor growth factor signaling. In agreement with these results, recent studies have shown that CAFs activate immune-suppressive pathways, promoting immune evasion and resistance to therapies [24,25,26].

### 4.3. RBX1-TNC Axis in Tumor Growth and Proliferation

Our investigation into the functional significance of RBX1 in PAAD cells revealed that RBX1 promotes cancer cell proliferation, colony formation, and spheroid growth. These in vitro findings were further corroborated by in vivo studies, where RBX1 knockdown led to reduced tumor size and weight in nude mice. These results suggest that RBX1 is a critical regulator of tumorigenesis and supports previous studies that have identified RBX1 as a key mediator of cell cycle regulation and tumor growth.

Additionally, we found that RBX1 regulates the expression of Tenascin C (TNC), an important ECM protein, in both cell culture and xenograft models. TNC overexpression partially rescued the tumorigenic potential of RBX1-depleted cells, indicating that RBX1 drives tumor growth through TNC-dependent mechanisms. This observation is consistent with recent studies linking TNC to poor prognosis in various cancers, including PAAD [27,28,29,30].

## 5. Conclusions

In summary, our study highlights the pivotal role of RBX1 in the TME, particularly in CAFs, and its contribution to PAAD progression. We demonstrate that RBX1 upregulation in CAFs facilitates ECM remodeling, immune evasion, and tumor growth, making it a potential therapeutic target for PAAD and possibly other cancers.

## Data Availability

The datasets used and/or analysed during the current study are available from the corresponding author on reasonable request.

## Supplementary Materials

The following supporting information can be downloaded at: https://www.mdpi.com/article/10.3390/cancers18061024/s1, Figure S1: Functional enrichment analysis, primary cell morphology, other E3 ligases and genotyping validation of genetically engineered mouse models. (A) Gene Ontology (GO) enrichment analysis of candidate genes. (B–I) Violin plots show the expression differences of E3 ligases with no significant differential expression in PAAD tumor (T) and normal (N) fibroblasts. (J) Representative bright-field image of primary pancreatic tumor-derived cells cultured in vitro. (K) Genotyping validation of genetically engineered mouse models (GEMMs) by PCR; Figure S2: Immunohistochemical staining of various samples from different patients and mice. (A) Immunohistochemical staining of patient samples. (B) Longitudinal study in KC mice. (C) Longitudinal study in KPC mice.
