# Supplementary material for: RBX1+ CAFs Drives Pancreatic Ductal Adenocarcinoma Progression Through Tenascin C Overexpression
Source: Cancers (Basel). 2026 Mar 22;18(6):1024. doi: 10.3390/cancers18061024 (PMC13025211; doi:10.3390/cancers18061024)
Supplement: Supplementary file 1 [file cancers-18-01024-s001.zip › cancers-4208388-supplementary figure.pdf]

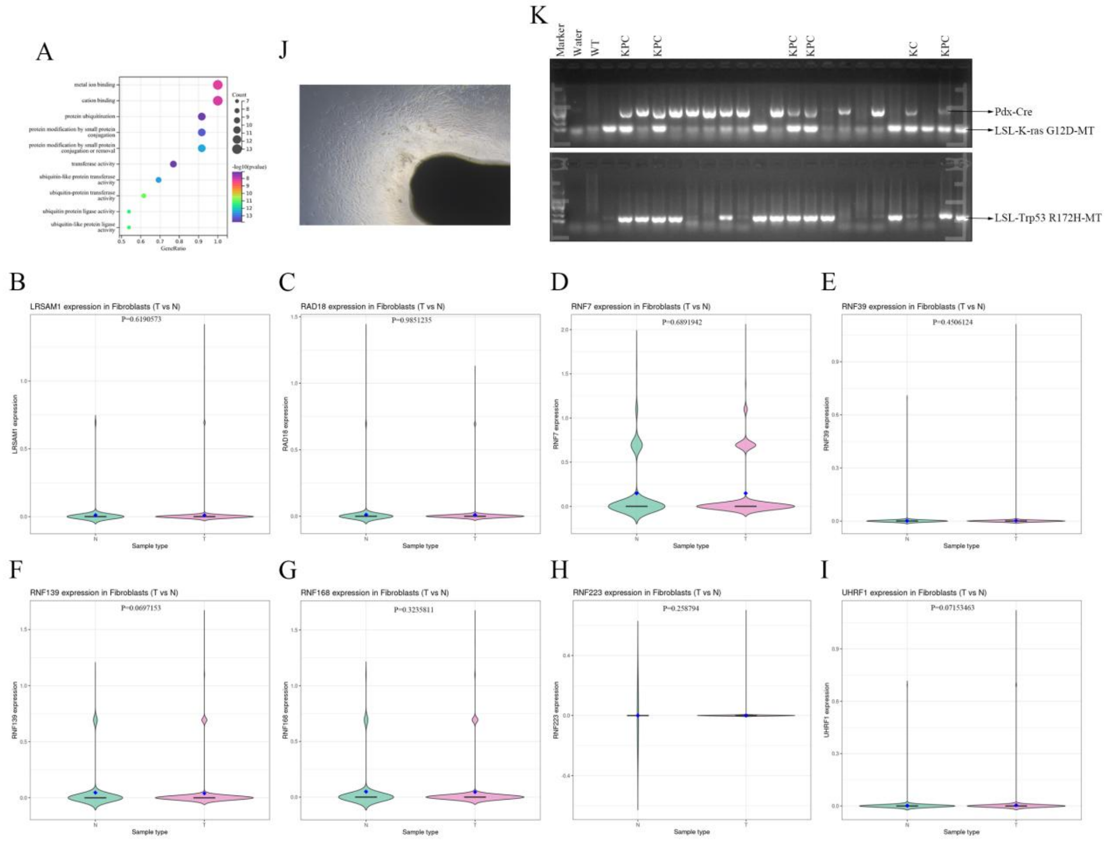

Figure S1. Functional enrichment analysis, primary cell morphology, other E3 ligases and genotyping validation of genetically engineered mouse models. (**A**) Gene Ontology (GO) enrichment analysis of candidate genes. (**B–I**) Violin plots show the expression differences of E3 ligases with no significant differential expression in PAAD tumor (T) and normal (N) fibroblasts. (**J**) Representative bright-field image of primary pancreatic tumor-derived cells cultured in vitro. (**K**) Genotyping validation of genetically engineered mouse models (GEMMs) by PCR.

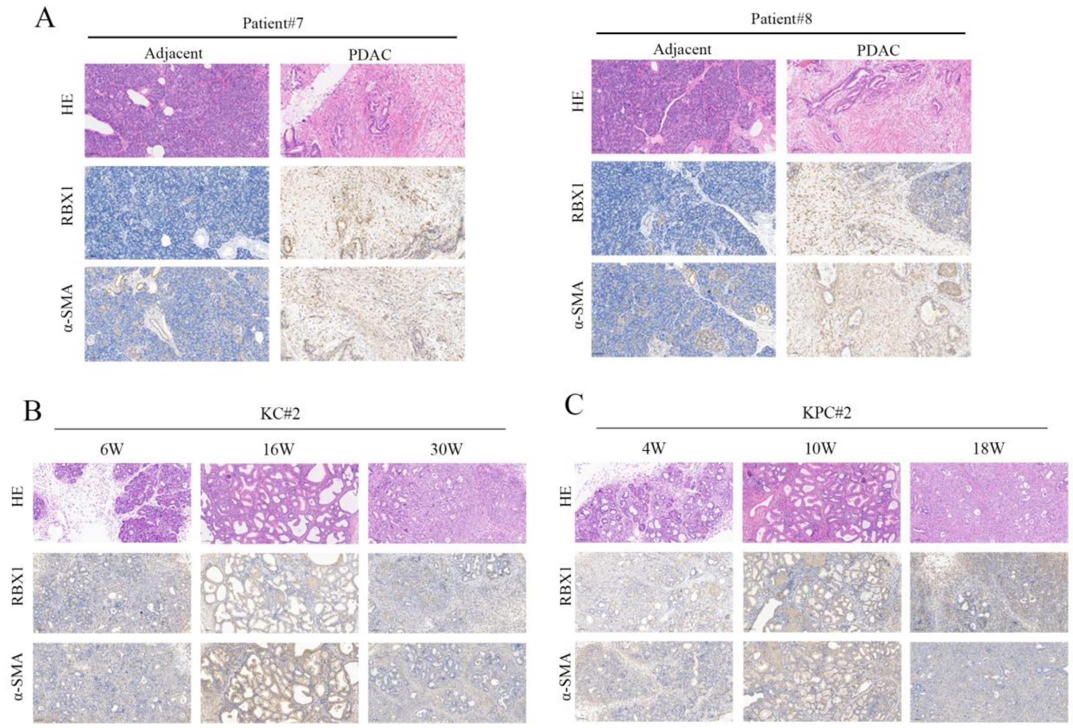

Figure S2. Immunohistochemical staining of various samples from different patients and mice. (**A**) Immunohistochemical staining of patient samples. (**B**) Longitudinal study in KC mice. (**C**) Longitudinal study in KPC mice.
