# Supplementary figures and images for: RBX1+ CAFs Drives Pancreatic Ductal Adenocarcinoma Progression Through Tenascin C Overexpression
Source: Cancers (Basel). 2026 Mar 22;18(6):1024. doi: 10.3390/cancers18061024 (PMC13025211; doi:10.3390/cancers18061024)

Figure 6C

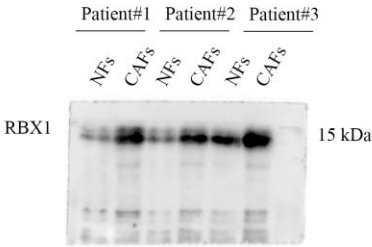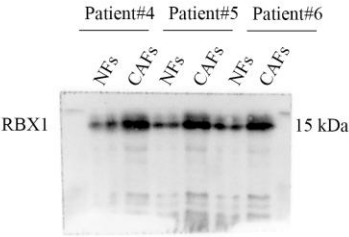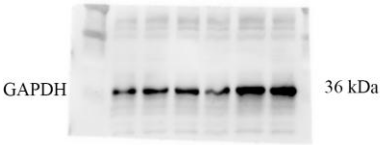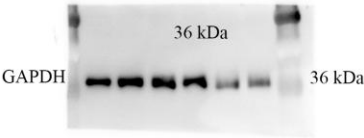

Figure 6F

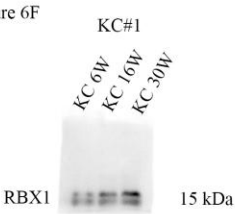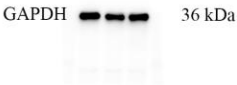

KC#2

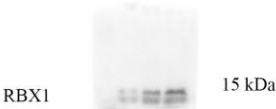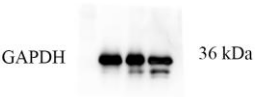

Figure 6I

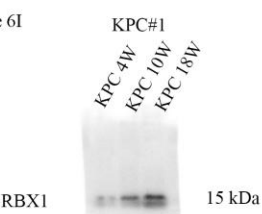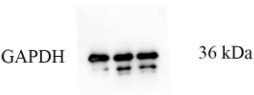

KPC#2

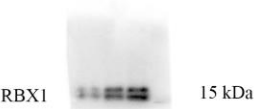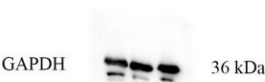

Figure 7A

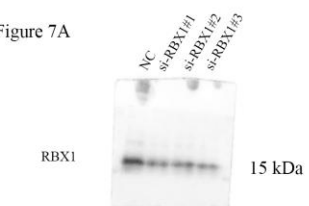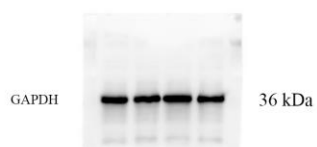

Figure 9A

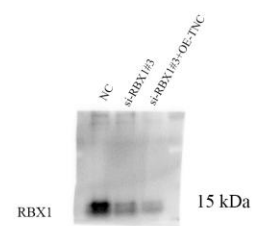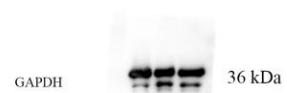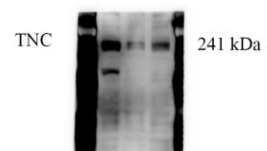

Supplement: Supplementary file 1 [file cancers-18-01024-s001.zip › Uncropped western blot figures.pdf]
